# The Impact of COVID-19 on Primary Care: A Scoping Review

**DOI:** 10.7759/cureus.33241

**Published:** 2023-01-02

**Authors:** Alam Khalil-Khan, Moien AB Khan

**Affiliations:** 1 Academic Unit of Primary Medical Care, The University of Sheffield, Sheffield, GBR; 2 Family Medicine, College of Medicine and Health Sciences, United Arab Emirates University, Al Ain, ARE; 3 Primary Care, North West London - National Health Service Provider, London, GBR

**Keywords:** healthcare, global, coronavirus, chronic diseases, redeployment of healthcare, virtual consultation, remote consulting, consultation covid, consultation styles, covid-19

## Abstract

The COVID-19 pandemic had a severe impact on various aspects of everyday life, including healthcare provision. The aim of the scoping review was to collate, summarize, and discuss this literature, in light of the impact COVID-19 had on Primary care. Preferred Reporting Items for Systematic Reviews and Meta-Analyses (PRISMA) four-stage process framework for reporting was followed. A total of 31 studies were included in this review. Based upon our review we found COVID-19 pandemic on Primary Care, has made significant effects on 1) service redesign, 2) long-term illness care provision, 3) healthcare staff well-being and 4) the post-pandemic future of Primary Care. The COVID-19 outbreak has demonstrated, how a pandemic can drastically change the process of healthcare provision within the community, as evidenced by the change in consultation styles with patients, the impact on the physical and mental wellbeing of health workers, a shift from isolated practice to teamwork, as well as the ability of healthcare workers to seek prompt help with their health. Studies have demonstrated progress in knowledge and experience gained by healthcare workers when tackling COVID-19, and how these can be implemented in possible future pandemics affecting Primary Care, however, further research is required within this sphere.

## Introduction and background

The COVID-19 pandemic began in December 2019 in China [1], and by March 2020, it was announced as a global emergency by the World Health Organization (WHO). In response, most countries declared mass lockdowns, to help contain the rapid spread of the virus within communities. The COVID-19 pandemic lockdowns affected multiple aspects of daily life globally, including food security, the global economy, education, tourism, hospitality, sports and leisure, and healthcare [2].

Primary Care system within countries is considered the first point of contact for people, with face-to-face consultations traditionally the way to assess and manage most healthcare problems, and as the foundation for the clinician-patient relationship. Primary Care plays an instrumental role in optimizing a country's response to a pandemic such as COVID-19. Gatekeeping and clinical responses were significantly enhanced by Primary Care, whereby people were identified and triaged for possible COVID-19 cases, making an early diagnosis, easing the anxiety of vulnerable people, and reducing the demand for hospital services. Due to strict control measures, including non-pharmaceutical interventions and hospital closings during periods of increased transmission, Primary Care has become increasingly important. Primary Care has also proved to be a more cost-effective response. In order to respond to COVID-19 at the local and national levels, a well-developed and well-equipped Primary Care system is essential. COVID-19 revealed that countries differed in terms of pre-existing universal health coverage, pandemic preparedness, and government and public support for public health measures, not only in public health, acute and long-term care but also in Primary Care [3,4].

Throughout the world, Primary Care physicians have been leading the pandemic response, and as a result of the additional workload, COVID-19 has had a considerable impact on Primary Care deliverance [5]. The pandemic has had both a positive and negative impact on Primary Care. A positive impact included the rapid incorporation of digital consulting tools [6], as well as the engagement of allied healthcare professionals to support medical staff within innovative roles to help bolster staffing levels. In addition, the reassessing and structuring of primary healthcare to aid in the effective delivery of Primary Care to patients was also implemented, and in certain cases, patient involvement in care decision-making was enhanced [7]. However, the pandemic has also displayed its negative impact on Primary Care, for example, in the form of disrupting chronic healthcare management for patients, and impact on healthcare staffing levels, leading to significant shortages and exhaustion within the workforce [8,9]. We aim to collate, summarize, and discuss this literature, and provide the readers with a review of the impact COVID-19 had on Primary care. We hope this review helps initiate further research, to help support and bolster Primary Care infrastructure globally. 

Methodology 

Our review discusses the impact of the COVID-19 pandemic on Primary Care. We have followed the PRISMA framework for reporting [10]. 

Information sources and search 

The research includes primary and secondary sources, mainly academic publications identified via professional databases including, PubMed, Scopus, and Google Scholar, as well as websites of relevant health establishments, departments, and ministries globally, that were linked to COVID-19 impact on Primary Care Research articles with the following headings “COVID-19” “coronavirus” “Global” “family medicine” “general practice” to help retrieve the medical literature for this review. Research articles dated from December 2019 to December 1, 2022 were used.

Inclusion criteria

The study relies on sources relevant to the COVID-19 pandemic and its effects on Primary Care. Documents included in the review were issued by a recognized national health authority and were specific to both Primary Care and COVID-19. All sources were limited to the English language. This review was extended to articles published from the time of the inception of COVID-19 in December 2019 to December 1, 2022. 

Study selection

The study selection process was undertaken by two reviewers who screened for abstracts, and full-text literature documents against the inclusion criteria. The process followed the PRISMA using the four-stage process [10].

Analysis procedures 

The literature was analyzed using both content analysis and the conceptual framework [11,12]. We undertook a descriptive summary of the literature, of all the included articles. Additionally, we provide a narrative synthesis of how COVID-19 has affected different Primary Care domains. 

Patient and public involvement 

Our review did not directly involve patients or the public in the conception of this study.

Results

**Figure 1:**
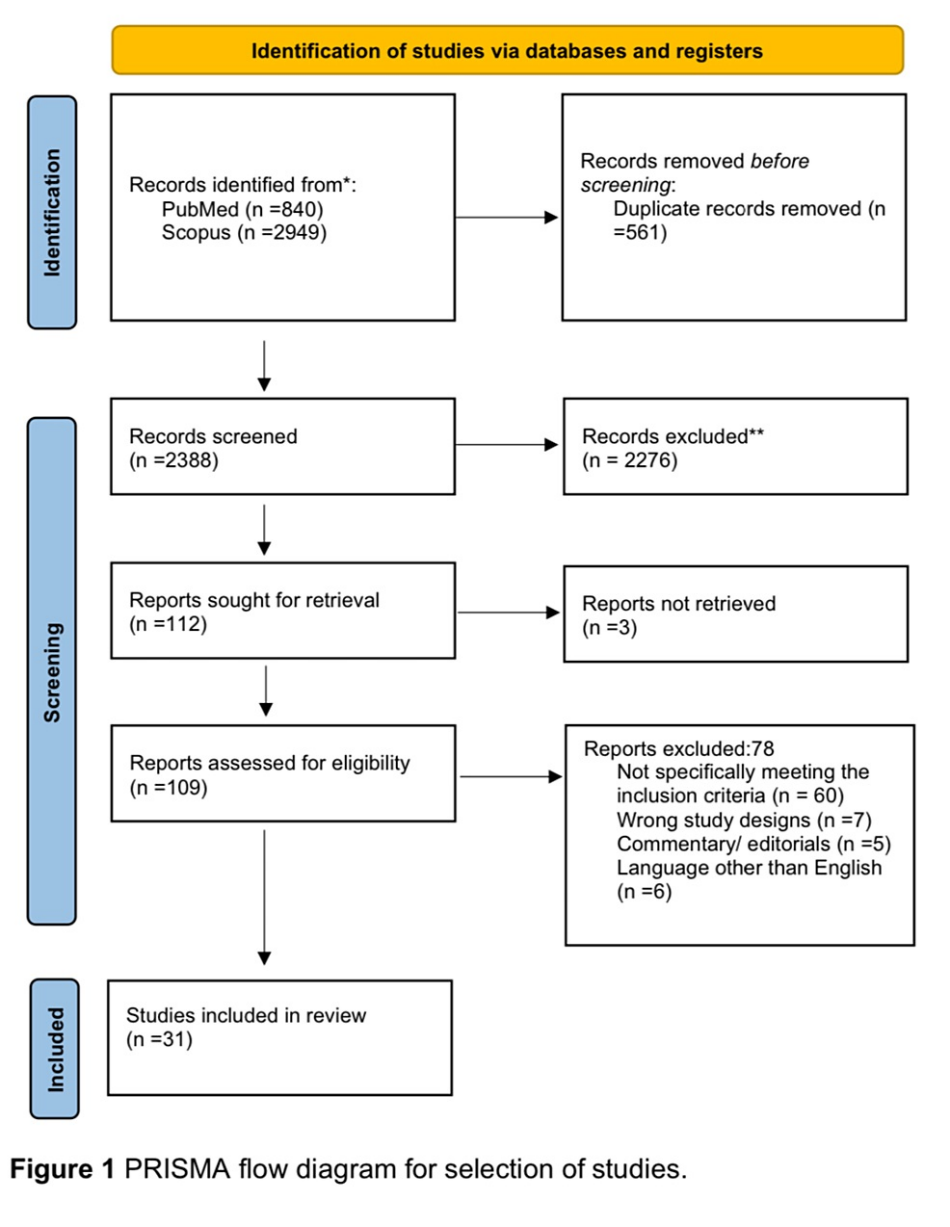
PRISMA flow diagram for selection of studies.

A total of 31 documents were retrieved from the literature that discussed the impact of COVID-19 on Primary Care. Figure 1 shows the PRISMA diagram of our results [13].

Whilst undertaking the search, documents meeting the inclusion criteria were found in Australia (n=3), Belgium (n=1), Canada (n=3), Netherlands (n= 1), United Arab Emirates (n=2), United Kingdom (n=12), USA (n=6), in addition to general global muti-centered articles (n=3) (including countries:- Australia, Canada, France, Germany, Israel, Netherlands, New Zealand, Sweden, Switzerland, the United Kingdom, United States, and continents:- Europe, Africa, Asia, Australia, and North and South America). These countries have established Primary Care structures. We have summarized the impact and effect of COVID-19 on Primary Care and the related outcomes (Table 1). The narrative synthesis identifies four key domains that were affected: changes in consultation styles, effects on long-term illness care provision, the impact of the pandemic on health care staff, and the future of Primary Care post-pandemic. The provision of Primary Care was hindered or facilitated by all of these factors.

**Table 1:** Impact of COVID-19 pandemic on Primary Care

Domain	Impact/Effect	Outcomes/Consequence
Consultation styles	Prevent the spread of the COVID-19 virus.	Rapid Incorporation of telemedicine. Disadvantage to virtual consultation, in the form of privacy, quality of care, sociodemographic safety.
Long-term illness care provision	Patient reluctant to consult in person with a practitioner. Difficulties in access to medical services for patients with long term health conditions.	Worsened in patient health status. Rise in patients who present with new diagnosis of long-term health conditions, which were potentially preventable.
Effect on healthcare staff	Difficult decision-making in times of uncertainty, in terms of resource allocation. Impact on physical and mental health needs of healthcare workers. Shortage of personal protection equipment. Fears of transmitting the virus to family members.	Highlights importance of a well-managed departments with supportive management structures. Depleted work force due to staff illness.
Future of family medicine post pandemic	Exposure of limitation of current family medicine practices. Redeploy staff to health care departments.	Telemedicine the new norm. Benefit of integrations digital platforms. Vast governmental investments, to create digital platforms. Limitation for technological disadvantaged patients. Future research opportunities

Our review based on content and thematic analyses found four major domains being affected in primary care as a result of COVID-19: effects on service redesign, impact on long-term illness care provision, effects on healthcare staff well-being, and the future of Primary Care post-pandemic.

Effects on Service Redesign

There have been some changes in service redesign found in our Scoping review, which has been tailored to prevent the spread of the COVID-19 virus (15, 16), in the form of rapid incorporation of telemedicine (14, 17-19). However, as with most medical innovations, there have been a few barriers to virtual consultation, in the form of privacy, eligibility, quality of the technology, quality of care, sociodemographic, and safety (20, 21, 37, 38).

Impact on Long-Term Illness Care Provision

There have been changes in regard to the difficulties patients have encountered when accessing medical services, in particular for patients with long-term health conditions (22-24, 27, 28). A shift was observed in patients’ reluctance in consulting with their Primary Care provider, in particular in those with long-term healthcare conditions. There is no doubt these patients are frequently classed as high risk or vulnerable to contracting illnesses such as a virus, and as a result, can have major health complications post-infection, and therefore these patients commonly chose not to consult Primary Care practitioners (25-28).

Effects on Healthcare Staff Well-Being

There has been some major change in healthcare workers' decision-making in times of uncertainty in particular when allocating resources within this challenging clinical situation (29, 30). This certainly has led to an impact on both the physical and mental health needs of healthcare workers, fears of transmitting the virus to family members, and lack of personal protection equipment, which has certainly led depleted workforce (31-36).

Future of Primary Care Post-pandemic

There has been some major shift within telemedicine initiatives that have rapidly been adopted within the norms of family medicine consultation and are deemed to remain a sensible approach to consulting patients within Primary Care. Some disadvantages, however, potentially lie within scenarios that require a safe environment, such as cases of domestic abuse (14, 17-21, 37, 38). The future of Primary Care also involves the integration and supplementation of allied healthcare roles, between physicians, nursing, and other healthcare staff (39). Massive governmental health initiatives and incentives are being adopted for a more advanced and efficient infrastructure (40, 41), but limit those who are at a technological disadvantage (42-44). 

## Review

The current scoping review found that COVID-19 has significantly affected primary care in four domains. There have been changes in consultation style and service redesign, provisions for long-term illness care, impacts on healthcare staff, and future practices in primary care.

During the COVID-19 pandemic, this traditional style of consultation required a rapid change from in-person to telemedicine consultations, either via video teleconferencing or telephone calls [14] to prevent the spread of the COVID-19 virus [15,16]. As mentioned by Murphy [14] a plan by NHS England was implemented to commit to Primary Care practices, by offering teleconsultations from April 2020 with the addition of video consultations from April 2021 (NHS England. The NHS Long Term Plan 2019). However due to the rapid progression of the pandemic, the UK government ordered Primary Care practitioners to undertake all consultations remotely, and thus the plan of teleconsultation, proposed by NHS England was brought into fruition earlier, to help reduce the spread of COVID-19. This change in the style of consultation was not limited to the UK, indeed within Europe, and in countries such as Norway, pre-pandemic digital consultations in Primary Care were around 5% which increased to almost 60% during the pandemic. In France, teleconsultations increased from around 10,000 per week before March, up to one million per week in April 2020, and finally, in Germany, it was estimated that 19,500 teleconsultations were performed in March, in comparison to 1,700 teleconsultations per month in early 2020. This trend was not limited to Europe, as globally it was noted that vast governmental investments, such as the Canadian federal government, putting in place new investments to create digital platforms, were introduced to either implement or expand the use of teleconsultation, in an attempted to limit disease spread [7].

Due to this rapid shift in consultation styles, patients were consulted by the practitioner via telephone and/or video [17-19]. Due to this shift in consultation styles, there was a requirement for the development of virtual platforms to help with such consultations, as such companies that offered these services expanded, allowing new consultation platforms to integrate with existing ones, at a favorable usage cost. Subsequently, the possible shortcoming of this type of consultation style was soon realized. As mentioned by Hardie et al. [20], there have been some barriers to virtual consultation, in the form of privacy, eligibility, quality of the technology, quality of care, sociodemographic, and safety, further echoed by Rosen et al., who discusses methods in which to minimize these effects, by potentially enhanced safety-netting, and training and support for staff, to help minimize risks of virtual consultation [21].

Prior to the COVID-19 pandemic, healthcare providers attempted to maintain reasonable access to medical services for patients who required care for their long-term health conditions. However, attempts to limit face-to-face contact and contain the infection during the pandemic, meant that patients had only limited access to healthcare providers, often having no physical contact for months. These strategies resulted in patients with long-term health problems, experiencing a delay in review and care. Patients with long-term health conditions such as cancer, heart disease, and mental health, saw a large decline in access to their Primary Care practitioners to help with these conditions, during the initial phases of the pandemic [22-24]. Primary Care physicians played a key role in the initial screening and chronic management of long-term health conditions. In addition to the lack of access to Primary Care physicians, it was also noted that patients themselves, were reluctant to be seen in primary, and secondary care due to the fear of contracting the virus. Indeed, patients with long-term healthcare conditions, are frequently classed as high risk or vulnerable to contracting illnesses such as a virus, and as a result, can have major health complications post-infection, and therefore these patients commonly chose not to consult Primary Care practitioners [25,26], resulting in further delay on review of their long-term chronic conditions. 

More recently, toward the current phase of the pandemic, there has been a shift in patient contact with their Primary Care physician, resulting in a rise in the number of patients consulting Primary Care practitioners, many of whom had not been followed up as usual during the peak of the pandemic, which has led to worsening of their condition. Disconcertingly, there has also been a rise in patients who present with a new diagnosis of long-term health conditions, which were potentially preventable if these patients had been reviewed and managed by a Primary Care practitioner earlier [27,28].

Decision-making during a non-pandemic environment can be in itself challenging for most healthcare workers. However, with the supplement of a pandemic, it has certainly added a new dimension to how decision-making in times of uncertainty is managed and has since been quite difficult for some healthcare workers when allocating resources within this challenging clinical situation [29,30]. This extra burden has certainly played a role in both the physical and mental health needs of healthcare workers [31]. A study by Gu et al. reveals that Primary Care practitioners felt overlooked and undervalued during the pandemic resulting in a negative impact [32], further highlighted by an article by Stephenson, where they mention Primary Care physicians suffering from burnout [33].

Issues raised by healthcare workers have been focused on the shortage of personal protection equipment, fears of transmitting the virus to family members and themselves, and the effects of a depleted workforce due to staff becoming unwell when contracting the infection, thus resulting in extra clinical stresses on the remaining staff. Reports of rare incidences of hostility and aggression from patients towards medical personnel have been noted. Effects on mental health, in particular depression, anxiety, and stress on healthcare workers, have been prevalent throughout this pandemic, and thus, it has been suggested to senior management to adopt supportive strategies as well as a motivational and encouraging environment, and one where training is provided should it be deemed necessary [34].

The pandemic has had physical health effects on healthcare workers that include exhaustion, sleep problems, headaches, and dehydration, whilst wearing protective equipment [35]. Most healthcare workers tend to be working within an environment where they, are at potentially higher risk of contracting the infection due to the job role, and where they do contract the illness, it places them at further risk of the chronic effects of COVID-19 infection, such as long covid, which could have a direct effect on their careers [34,36]. This again reiterates the importance of a well-managed department, which possess a supportive management structure and support from the local occupational health department. It also relies on the self-awareness of the healthcare worker, to understand their limitations and when to promptly seek help and advice.

Healthcare workers are discovering new intelligence on how to tackle the pandemic within their community, and it is hoped this experience will help prevent or minimize serious untoward effects of future pandemics globally. Telemedicine initiatives have rapidly been adopted within the norms of family medicine consultation and are deemed to remain a sensible approach to consulting patients, especially those with high-risk health conditions, where potential remote patient monitoring, can be implemented within Primary Care [37]. Some disadvantages potentially lie within scenarios that patients potentially face, such as domestic violence, in which a face-to-face consultation, would be preferred, where a safe environment is employed for these patients to discuss their difficulties [38].

Future of Primary Care also involves the integration and supplementation of allied healthcare roles, between physicians, nursing, and other healthcare staff, which were observed during the peak of the pandemic, and are set to develop further, to assist in the development and integration of a holistic approach to patient care [39]. The COVID-19 pandemic has certainly put additional pressure on pre-existing difficulties faced with integrating care pathways for patients with major social and healthcare needs, it is therefore imperative this is taken into consideration when integrating roles. The pandemic has provided an opportunity to reflect on how to address these challenges within a new era of healthcare deliverance. 

Knowledge and experience gained by healthcare workers when tackling COVID-19, have highlighted additional gaps in knowledge and have certainly stressed in importance of further research within this discipline. Focus on the implementation of robust primary healthcare which provides comprehensive and preventative measures. Such an approach has demonstrated how resilient infrastructure has been since developed and remains the focus for future public health emergencies. The use of teleconsultations has thus far proved beneficial, demonstrating ways of employing technology in health care that can be developed further with governmental health initiatives and incentives creating foundations for a more advanced and efficient infrastructure [40,41], this will certainly be further enhanced, in particular when consulting patients with long term health conditions or those who do not have access to the virtual world, that request traditional face to face contact with their Primary Care providers [42-44]. Recent events have highlighted to healthcare workers, that effects on Primary Care is not just confined to solely pandemic-related incidence and can also occur during other misfortunes such as financial crisis, natural disasters, and acts of major terrorism. Post-pandemic Primary Care providers have identified these factors and have implemented their experience acquired post-pandemic to reduce negative impacts potentially caused by such incidents.

Strengths and limitations

This is the first systematic scoping review, to our knowledge, to incorporate an overview of the impact of COVID-19 on Primary Care. We have included literature from a variety of countries for strategies in adapting their approach to Primary Care within a COVID-19 era. The present study has limitations, such as limited evidence from previous pandemics related to its effects on Primary Care. Nevertheless, in our review, we followed clear objectives with inclusion criteria. Furthermore, our study calls for further research into managing strategies, when tackling COVID-19, and the application of such methods. Finally given the nature of the COVID-19 pandemic, this review was primarily related to countries where COVID-19 had an effect on the Primary Care structure, and thus did not include certain countries that do not have such infrastructures. 

## Conclusions

The COVID-19 outbreak has demonstrated globally, how a pandemic can drastically change the way healthcare is provided within Primary Care. It has drastically changed the way in which we consult our patients and has highlighted both advantages and disadvantages of face-to-face and remote consultations. It has indeed enforced a rapid change in the way patients are referred and subsequently followed up. In addition, the COVID-19 pandemic has had a significant impact on healthcare workers, both with their physical and mental health, and has shown the importance of good managerial support, and reinforced the importance of teamwork, as well as the ability of healthcare workers to seek prompt help with their health, if it is required.
